# Complex spatial light modulation capability of a dual layer in-plane switching liquid crystal panel

**DOI:** 10.1038/s41598-022-12292-4

**Published:** 2022-05-18

**Authors:** Seong-Woo Jang, Wonwoo Choi, Soobin Kim, Jonghyun Lee, Sehwan Na, Sangwon Ham, Juseong Park, Hoon Kang, Byeong-Kwon Ju, Hwi Kim

**Affiliations:** 1 Display and Nanosystem Laboratory, Department of Electrical of Engineering, Korea University, 145, Anam-ro, Seongbuk-gu, Seoul, 02841 Republic of Korea; 2 Department of Electronics and Information Engineering, Korea University, Sejong Campus, Sejong, 30019 Republic of Korea; 3 LG Display, E2 Block LG Science Park, 30, Magokjungang 10-ro, Gangseo-gu, Republic of Korea

**Keywords:** Engineering, Optics and photonics

## Abstract

Complex spatial light modulator (SLM), which can simultaneously control the amplitude and phase of light waves, is a key technology for wide-range of wave-optic technologies including holographic three-dimensional displays. This paper presents a flat panel complex spatial light modulator that consists of dual in-plane switching liquid crystal panels with double-degrees of freedom of voltage inputs. The proposed architecture features single-pixel level complex light modulation enabling complex light modulation in entire free space, which is most contrast to conventional macro-pixel based complex modulation techniques. Its complex light modulation capability is verified with theoretical simulation and experimental characterization, and a three-dimensional holographic image reconstruction without conjugate noise. It is believed that the proposed flat panel complex SLM can be an essential device for a wide range of advanced wave optic technologies.

## Introduction

Synthesis of wave field is a fundamental technology. The spatial light modulator (SLM), an essential device that directly modulates wavefront of light wave, provides a way of wave field synthesis and modification at the design level. The digital holography technology such as holographic imaging and holographic display are the representative field that get benefit from the SLM technology^1–6^. Moreover, the SLMs have been extensively used to wave optic technologies such as beam steering^7^, optical communications^8,9^, advanced microscopy and biomedical imaging^10,11^.

The modulation performance of SLMs sets a fundamental limitation to the overall performance of wave-optic based technologies. Achieving the controllability of wave optic field distribution with high efficiency and low noise is highly desirable in common for the transmission-type SLM and reflection-type SLM^12–17^. The development of SLM technology have been raised in variety of directions such as transmission-type liquid crystal panel^1,2,11,18,19^, reflection-type liquid crystal on Silicon (LCoS)^5,8,20^, reflection-type digital micro-mirror device (DMD)^4,10^, and recently emerging active metaphotonics SLM^21–23^. Though a number of SLM approaches with amplitude or phase-only modulation have been introduced, most suffer from several types of noise problem, such as DC and conjugate noises^12,24,25^. A number of research have attempted to overcome these issues. Some approaches implemented the additional systems to filter the noises^26,27^ yet most made the system less efficient or bulky. Other well-known approaches are encoding additional computer-generated hologram (CGH) design algorithm to a phase-only SLM^25,28,29^, but they made the system time-consuming or offer the modulation at only restricted field. It has been agreed that the fundamental solution to overcoming this problem is genuine complex light modulation, which means simultaneous control of both the amplitude and phase in a single pixel level.

In particular, the complex SLM, which modulates the amplitude and phase of the incident light, is highly applicable to numerous engineering fields with display applications. The complex SLM is crucial for digital three-dimensional (3D) holographic displays^30^. Recently, augmented reality based advanced holographic vision for metaverse or mixed reality, and emerging optical computing technology such as all optical neural networks have become the promising application fields of complex SLM technology^21,31–34^.

However, a practical solution to complex SLM has been a challenging problem for several decades. A consideration should be given to a challenge to make a single-panel LC SLMs with complex modulation characteristics because at least two independent control degrees of freedom, such as two independently controllable voltage electrodes, are necessary to modulate the amplitude and phase of light separately. The integration of two independent voltage electrodes into a single LC panel structure is not allowed by current LCD manufacturing infrastructure.

One approach to achieve complex light modulation is using single plane macro-pixel structures, which is using in-plane multiple pixels to compose a certain amplitude-phase value^14,16^. The Idea is that two or more pixels with different phase values are coherently interfered to achieve complex modulation that single pixel is unable to achieve. However, this method enables complex modulation within only certain region of interests but unable to realize the complex modulation for entire field, which includes both near-field and far-field. Moreover, the spatial light modulators with ultra-high resolution are required to achieve macro-pixel structure, since it needs multiple pixels for a single amplitude-phase unit. For these reasons, the applications for single-plane macropixel structures are limited.

The second approach to achieve transmissive type complex SLM is using the architecture of amplitude-phase dual-layer liquid crystal (LC) SLM^2,35^. The phase-SLM is attached to the amplitude-SLM so as to sequentially manage the amplitude or phase of the transmissive incident light. However, designing and fabricating perfectly pixel-matched amplitude and phase transmissive SLMs is unattractive in terms of design, fabrication, and cost. The amplitude-phase dual-layer LC structure is inefficient, concerning that the architectures of the phase-LC SLM and the amplitude-LC SLM cannot be the same in terms of LC cell gap and polarization configuration. The polarization layer should be placed between the amplitude-LC panel and the phase-LC panel leading to thick and expensive architecture.

Moreover, our concern is about the phase-SLM construction. Specifically, the layered architecture of the amplitude-panel and phase-panel is not available for the in-plane switching (IPS) LC panels. The manufacturing infrastructure of the IPS LCD has been well developed. The IPS LC mode is popularly used for the amplitude modulation of light and the IPS LCD is a prevailing LCD in the world-wide commercial display market for large viewing angle and sufficiently fast response. However, it is well known that the IPS LC mode has poor phase modulation characteristics. A theory supports the maximum phase modulation dynamic range of the single IPS LC mode being less than π (rad.). That maximum π phase modulation condition induces a severe degradation in the amplitude modulation characteristics in the IPS LC mode. Therefore, the popular IPS LC mode cannot be applied to the amplitude-phase dual layer architecture. Thus, the generally accepted opinion is that the IPS LC mode is only useful for amplitude modulation and neither for phase modulation nor for complex modulation.

In this paper, to overcome this limitation of the IPS-LC mode for the complex SLM, we propose a novel dual-layer complex SLM architecture featuring two perfectly identical in-plane switching (IPS) LC layers. We present a theory of using two identical IPS panels to achieve a perfect complex light modulation function and experimental demonstration of the pixel-level complex light modulation.

## Theory

**Figure 1:**
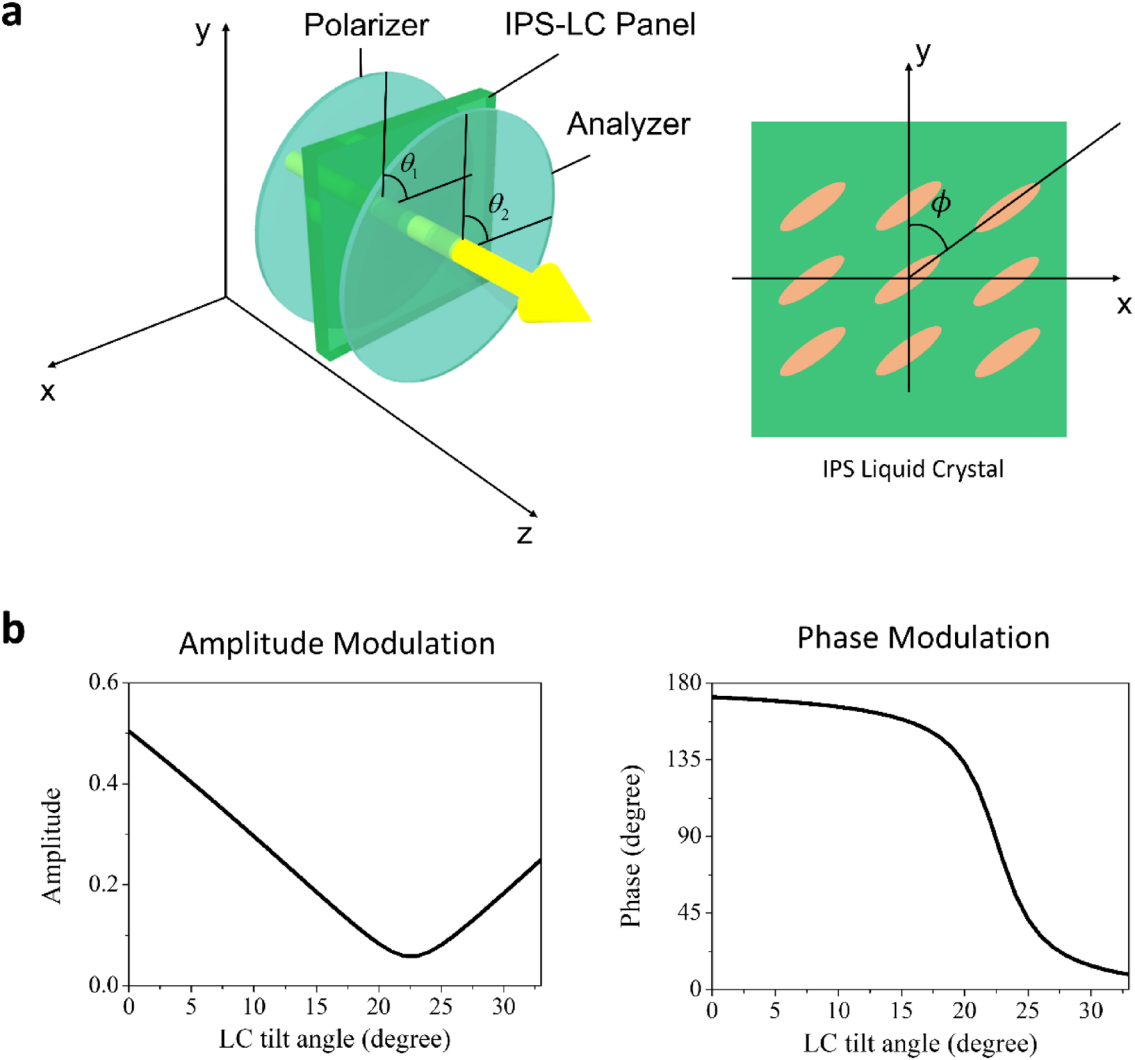
(**a**) Schematics of the single pixel of the conventional single-layer IPS-LC SLM (left panel) and its IPS-LC layer (right panel). (**b**) The amplitude (left panel) and phase modulation (right panel) characteristics of the single-layer IPS-SLM.

Figure 1a illustrates the schematic of the conventional SLM with an IPS LC panel. As the incident light passes through the LC panel, the orientation of the liquid crystal modulates the amplitude and the phase of the output light beam.

Let the polarization angles of the polarizer and the analyzer be denoted by *θ*_1_ and *θ*_2_, respectively, and the LC tilt angle of each IPS panel be *ϕ*. The phase retardation factor of the single IPS LC cell is then given by $\Gamma = 2\pi \left( {n_{e} - n_{o} } \right)d/\lambda$, where *λ* is the wavelength of the incident light, *d* is the cell gap of the LC layer, and *n*_*e*_ and *n*_*o*_ are the refractive indices of the extraordinary and the ordinary axes of the liquid crystal used in the IPS panel. The single pixel transmittance of the IPS-LC SLM can be modeled by a simple Jones matrix describing triple matrix multiplication^36–42^,

1 $$T\left( {\phi ;\theta_{1} ,\theta_{2} ,\Gamma } \right) = P\left( {\theta_{2} } \right)L\left( {\phi ,\Gamma } \right)P\left( {\theta_{1} } \right),$$

where *P*(*θ*) and *L*(*ϕ*, Γ) are the Jones matrices of the polarizer and IPS-LC layer, respectively. The Jones matrix for polarizer and analyzer, *P*(*θ*), is given by

2 $$P\left( \theta \right) = \left( {\begin{array}{*{20}c} {\cos \theta } & { - \sin \theta } \\ {\sin \theta } & {\cos \theta } \\ \end{array} } \right)\left( {\begin{array}{*{20}c} 1 & 0 \\ 0 & 0 \\ \end{array} } \right)\left( {\begin{array}{*{20}c} {\cos \theta } & {\sin \theta } \\ { - \sin \theta } & {\cos \theta } \\ \end{array} } \right).$$

The Jones matrix of an IPS LC layer with an LC tilt angle *ϕ* and the phase retardation Γ is represented as

3 $$L\left( {\phi ,\Gamma } \right) = e^{{j\frac{2\pi }{\lambda }n_{o} d}} \left( {\begin{array}{*{20}c} {\cos \phi } & { - \sin \phi } \\ {\sin \phi } & {\cos \phi } \\ \end{array} } \right)\left( {\begin{array}{*{20}c} {e^{j\Gamma } } & 0 \\ 0 & 1 \\ \end{array} } \right)\left( {\begin{array}{*{20}c} {\cos \phi } & {\sin \phi } \\ { - \sin \phi } & {\cos \phi } \\ \end{array} } \right).$$

The amplitude and phase modulation characteristics of the single IPS-LC SLM is calculated with the model of Eq. (1). Figure 1b presents the calculated amplitude and phase modulation characteristics of the conventional IPS-LC SLM with *θ*_1_ = 110° and *θ*_2_ = 0°, in which the maximum range of phase modulation is obtained. The parametric study shows that the phase modulation range does not exceed 180-degree even at the best condition. As mentioned above, it means that the IPS-LC mode is not appropriate for the amplitude-phase dual-layer SLM architecture.

**Figure 2:**
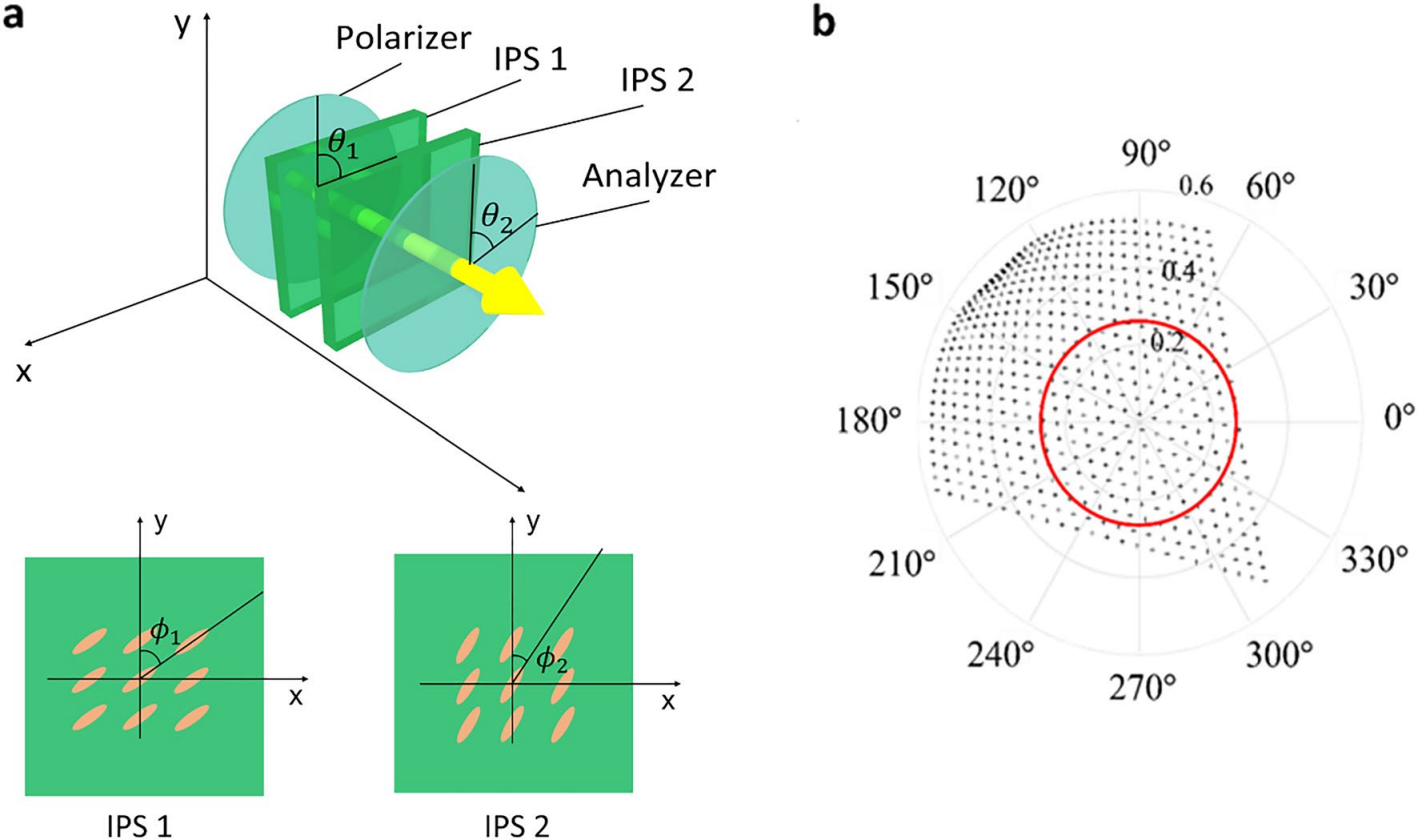
(**a**) Schematics of the single pixel of the proposed dual-layer IPS-LC SLM (upper panel) and its first and second IPS-LC layers (lower panel). (**b**) Simulated complex light modulation range of the dual-layer IPS-LC SLM. The red circle indicates the amplitude of 0.26.

Figure 2a illustrates the schematic structure of the proposed dual-layer IPS-LC SLM. The proposed device consists of two IPS-LC layers placed sequentially between a polarizer and an analyzer. Each IPS-LC layer operates as a dynamic wave plate. In practice, two completely identical IPS-LC panels are finely aligned and attached in parallel. The degree of freedom necessary for complex light modulation is the LC tilt angle of each IPS panel, *ϕ*_1_ and *ϕ*_2_.

The Jones matrix modeling of the dual-layer IPS LC layers describes the transmittance of the single pixel of the dual-layer IPS-LC SLM by four-matrix multiplication model

4 $$T\left( {\phi_{1} ,\phi_{2} ;\theta_{1} ,\theta_{2} ,\Gamma } \right) = P\left( {\theta_{2} } \right)L\left( {\phi_{2} ,\Gamma } \right)L\left( {\phi_{1} ,\Gamma } \right)P\left( {\theta_{1} } \right),$$

For a linearly *x*-directional polarized incident light, we can set the polarization axis angle to *θ*_1_ = 0 without loss of generality, which makes *P*(*θ*_1_) = (1, 0). After some manipulation of Eq. (4), the electric field after the analyzer at the x-polarized light incidence is obtained as we have the total Jones matrix representation as the following form:

5 $$\left( {\begin{array}{*{20}c} {E_{x} } \\ {E_{y} } \\ \end{array} } \right) = e^{{j\frac{4\pi }{\lambda }n_{0} d}} \left( {\begin{array}{*{20}c} {e^{j2\Gamma } \cos \theta_{2} A_{1} + e^{j\Gamma } \cos \theta_{2} A_{2} + \cos \theta_{2} A_{3} } \\ {e^{j2\Gamma } \sin \theta_{2} A_{1} + e^{j\Gamma } \sin \theta_{2} A_{2} + \sin \theta_{2} A_{3} } \\ \end{array} } \right),$$

where *A*_1_, *A*_2_, and *A*_3_ are

6 $$A_{1} = (\cos \theta_{2} \cos \phi_{1} + \sin \theta_{2} \sin \phi_{1} )\cos \phi_{2} \cos \Delta \phi ,$$

7 $$A_{2} = (\cos \theta_{2} \sin \Delta \phi + \sin \theta_{2} \cos \Delta \phi )\sin \Delta \phi ,$$

8 $$A_{3} = (\cos \theta_{2} \sin \phi_{1} - \sin \theta_{2} \cos \phi_{1} )\sin \phi_{2} \cos \Delta \phi ,$$

and Δ*ϕ* is

9 $$\Delta \phi = \phi_{2} - \phi_{1} .$$

We take the linear polarization component *U* along the polarization axis of the analyzer.

10 $$\left( {\begin{array}{*{20}c} U \\ V \\ \end{array} } \right) = \left( {\begin{array}{*{20}c} {\cos \theta_{2} } & {\sin \theta_{2} } \\ { - \sin \theta_{2} } & {\cos \theta_{2} } \\ \end{array} } \right)\left( {\begin{array}{*{20}c} {E_{x} } \\ {E_{y} } \\ \end{array} } \right) = e^{{j\frac{4\pi }{\lambda }n_{0} d}} \left( {\begin{array}{*{20}c} {e^{j2\Gamma } A_{1} + e^{j\Gamma } A_{2} + A_{3} } \\ 0 \\ \end{array} } \right).$$

Here, our main finding about *U* reveals that, with Γ = 2*π*/3, the characteristics of the transmitted light is represented by the form of a three-phase-amplitude complex modulation:

11 $$U\left( {\phi_{1} ,\phi_{2} ;\Gamma } \right) = A_{1} \left( {\phi_{1} ,\phi_{2} } \right)e^{j4\pi /3} + A_{2} \left( {\phi_{1} ,\phi_{2} } \right)e^{j2\pi /3} + A_{3} \left( {\phi_{1} ,\phi_{2} } \right),$$

where, for simplicity, the constant $\exp \left( {j4\pi n_{o} d/\lambda } \right)$ is omitted. With this three-phase formula, a particular amplitude and phase modulation is accomplished by modulating the real variables, *A*_1_, *A*_2_, and *A*_3_. In general, the phase retardation Γ can be a multiple of 2*π*/3. A parametric study for *θ*_1_ and *θ*_2_ to find the optimal complex modulation condition reveals that the pair of the polarizer and analyzer with *θ*_1_ = 0° and *θ*_2_ = 125° achieves the widest complex modulation dynamic range.

Figure 2b presents the asymmetric full complex modulation range in the complex plane. It includes a red-colored circle indicating a maximum amplitude 0.26, which means that the full complex modulation within 6.7 percent light modulation efficiency is obtained in the proposed dual-layer IPS SLM. The maximum modulation amplitude is denoted by *η*_max_ such that *η* ≤ *η*_max_. Given a complex modulation value, $\eta \exp \left( {j\psi } \right)$, we can find the LC tilt angles (*ϕ*_1_, *ϕ*_2_) by solving the nonlinear equation system of the real and imaginary parts of Eq. (11) under the constraint of *η* ≤ *η*_max_,

12 $$\left( {\phi_{1}^{\prime } ,\phi_{2}^{\prime } } \right)\mathop { = \arg \min }\limits_{{\left( {\phi_{1} ,\phi_{2} } \right)}} \left| {F_{R} \left( {\phi_{1} ,\phi_{2} } \right) + F_{I} \left( {\phi_{1} ,\phi_{2} } \right)} \right|,$$

where *F*_*R*_(*ϕ*_1_, *ϕ*_2_) and *F*_*I*_(*ϕ*_1_, *ϕ*_2_) are defined by the discrepancies of the real and imaginary parts,

13 $$F_{R} \left( {\phi_{1} ,\phi_{2} } \right) = \left| {A_{1} \left( {\phi_{1} ,\phi_{2} } \right)\cos \left( {4\pi /3} \right) + A_{2} \left( {\phi_{1} ,\phi_{2} } \right)\cos \left( {2\pi /3} \right) + A_{3} \left( {\phi_{1} ,\phi_{2} } \right) - \eta \cos \psi } \right|^{2} ,$$

14 $$F_{I} \left( {\phi_{1} ,\phi_{2} } \right) = \left| {A_{1} \left( {\phi_{1} ,\phi_{2} } \right)\sin \left( {4\pi /3} \right) + A_{2} \left( {\phi_{1} ,\phi_{2} } \right)\sin \left( {2\pi /3} \right) - \eta \sin \psi } \right|^{2} .$$

We used the fsolve routine of MATLAB to specify the IPS-LC tilt angles (*ϕ*_1_, *ϕ*_2_). As a specific case, the complete black is obtained by setting $F_{R} \left( {\phi_{1} ,\phi_{2} } \right) = F_{I} \left( {\phi_{1} ,\phi_{2} } \right) = 0$, which is represented as

15 $$\left( { - 1/2} \right)A_{1} \left( {\phi_{1} ,\phi_{2} } \right) + \left( { - 1/2} \right)A_{2} \left( {\phi_{1} ,\phi_{2} } \right) + A_{3} \left( {\phi_{1} ,\phi_{2} } \right) = 0,$$

16 $$A_{1} \left( {\phi_{1} ,\phi_{2} } \right)\left( { - \sqrt 3 /2} \right) + A_{2} \left( {\phi_{1} ,\phi_{2} } \right)\left( {\sqrt 3 /2} \right) = 0,$$

This is equivalent to the condition of $A_{1} \left( {\phi_{1} ,\phi_{2} } \right) = A_{2} \left( {\phi_{1} ,\phi_{2} } \right) = A_{3} \left( {\phi_{1} ,\phi_{2} } \right)$, meaning that, for the black mode, *A*_1_, *A*_2_, and *A*_3_ do not need to be zero, but can be non-zero values. It should be noted that the complex modulation mechanism is proven in a single pixel level of the dual-layer LC-IPS SLM enabling complex light modulation in entire free space. Therefore, we have proven that the industrial IPS panel technology can be successfully utilized to realize the complex SLM.

The phase retardation Γ is a critical parameter for determining the shape of the entire complex modulation dynamic range and the maximum modulation efficiency. The variation in Γ can be induced by several physical parameters such as deviations in operating wavelength, cell-gap deviation, and incidence angle of the light source and other LC panel geometries. Further theoretical simulation on the complex modulation efficiency and characteristics of the dual-layer IPS SLM is carried out in the Supplementary Information. For a fast addressing, the numerical data of (*ϕ*_1_, *ϕ*_2_) for the given discrete sampled complex modulation value $\eta_{m} \exp \left( {j\psi_{m} } \right)$ can prepared in the lookup table, and a numerical interpolation is used to accurately extract (*ϕ*_1_, *ϕ*_2_) for a given $\eta \exp \left( {j\psi } \right)$ based on the modulation look-up table.

## Experiments

### Measurement of complex spatial light modulation characteristics

**Figure 3:**
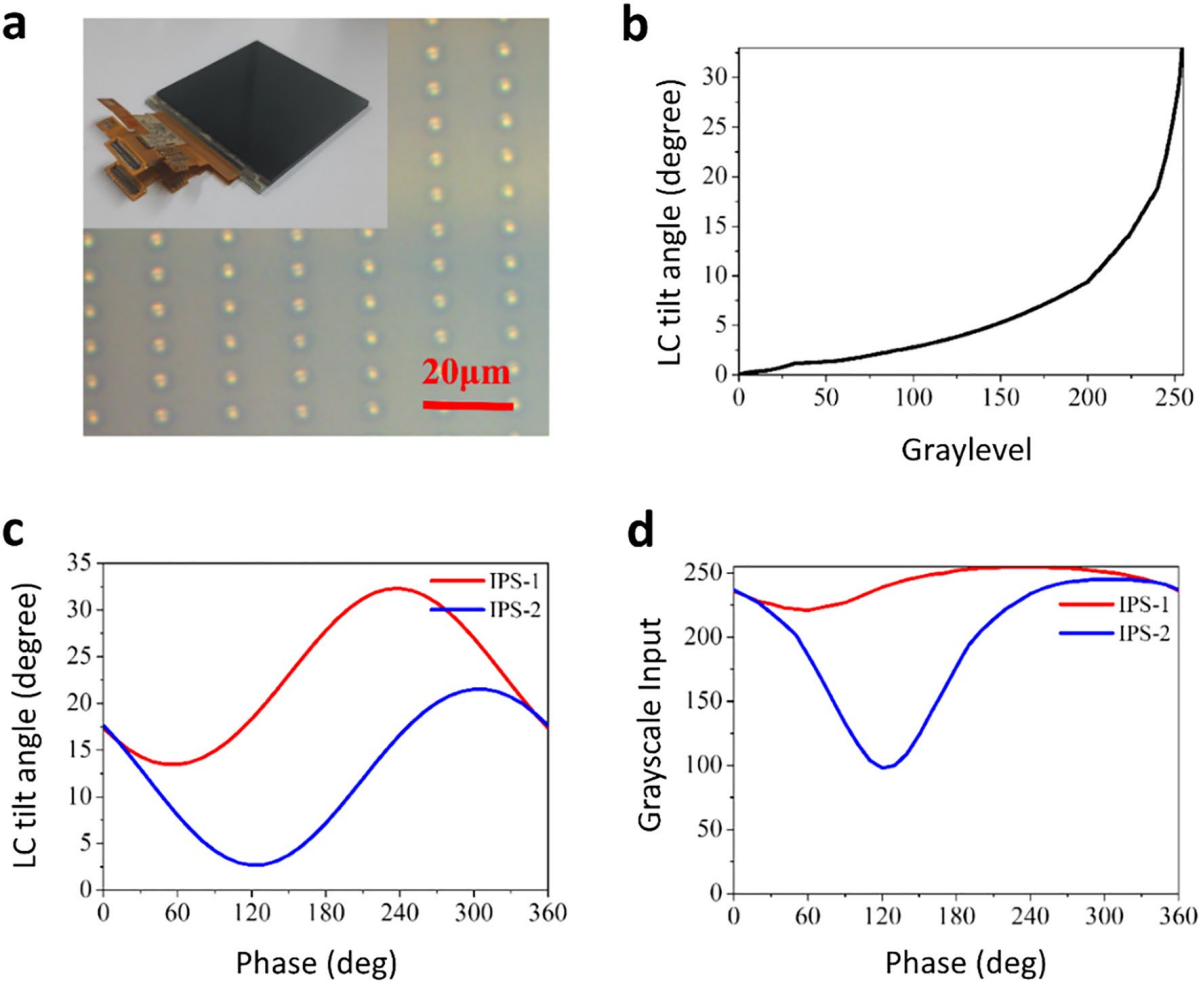
(**a**) Fabricated dual-layer IPS panel (inset) and its optical microscope image. (**b**) Relationship between the input gray level and the IPS-LC tilt angle. (**c**) Tilt angles of IPS-1 and IPS-2 in the dual-layer IPS panel required for 360-degree phase modulation and (**d**) grayscale inputs required for IPS-1 and IPS-2 for 360-degree phase modulation.

The actual dual-layer IPS panel is fabricated for the experiment to prove the proposed complex modulation theory in practice. Two identical IPS panel is fabricated, and then attached together with precisely aligned condition. Figure 3a shows the fabricated dual-layer IPS device and its photo image taken by optical microscope. Table 1 shows the basic parameters of the developed IPS display, and Fig. 3b shows the gray level input for certain LC tilt angles ranged from 0(deg.) to 35(deg.), for the IPS-LC panel used in the experiment. For the experimental demonstration, the required input for the full modulation is found based on the simulation result. For achieving Γ = 4*π*/3 instead of 2*π*/3, we set the LC panel cell gap d to 2.8 μm. The retardation Γ = 4*π*/3 is a multiple of 2*π*/3, and therefore it maintains the three-phase formula and simultaneously sets the cell gap close to industrially acceptable value. The required LC tilt angle pairs for 360-degree phase modulation and the corresponding operating voltage inputs are calculated by solving Eqs. (13)-(14). Figure 3c shows *ϕ*_1_ and *ϕ*_2_ producing 360-degree phase modulation with a constant amplitude. Conversely, we can obtain the amplitude modulation with a constant phase. From this data, the required pairs of gray level inputs for full complex modulation in Fig. 2b are found. The grayscale inputs presented in Fig. 3d are obtained for 360-degree full-phase modulation.

**Table 1:** The structural parameters of the fabricated IPS device.

Display resolution	Cell size	Aperture ratio	Cell gap d	Ordinary refraction index *n*_*o*_	Extraordinary refractive index *n*_*e*_
2560 × 1600	16.4 μm × 8.2 μm	7.15%	2.8 μm	1.4902	1.6197

**Figure 4:**
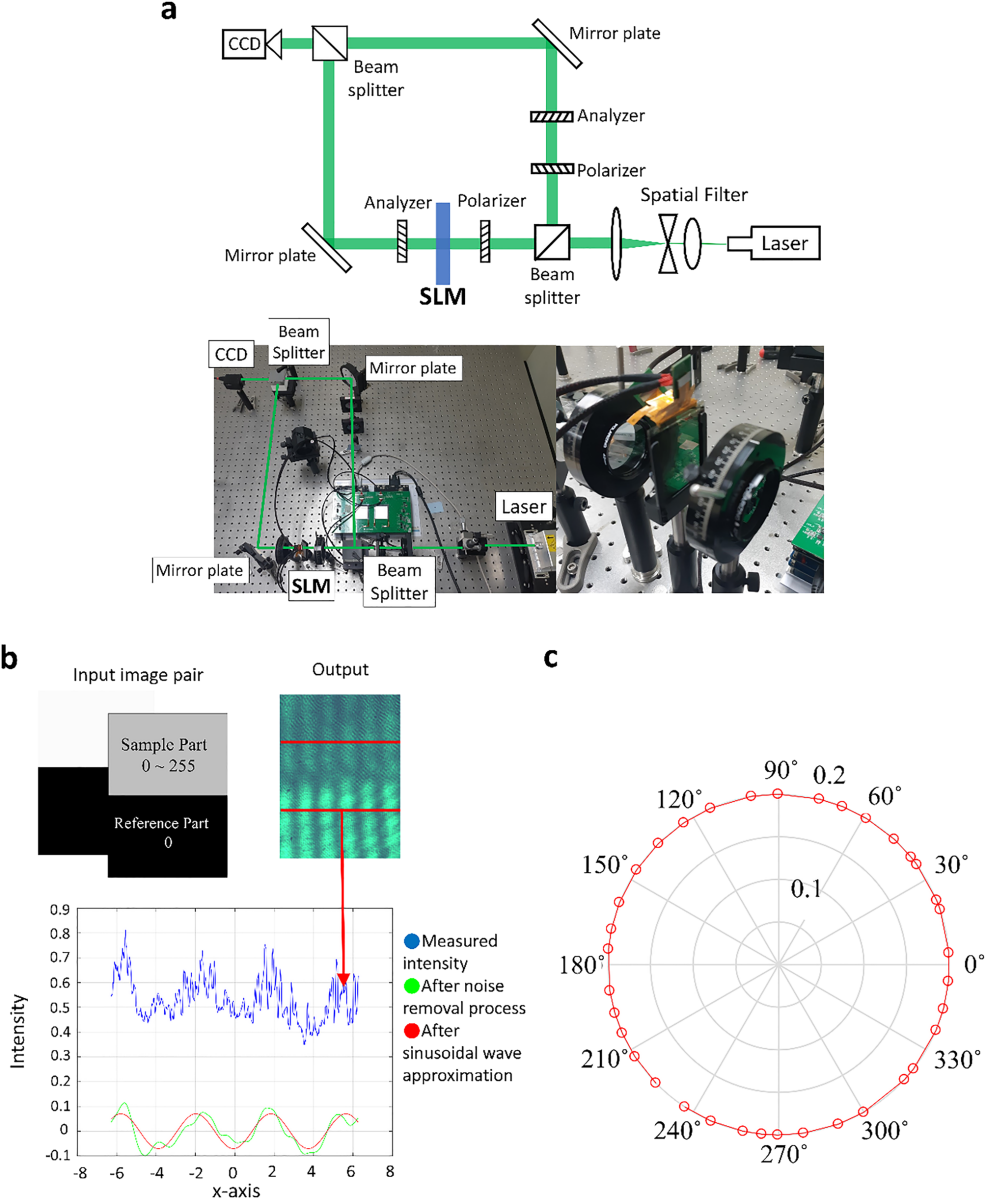
(**a**) Schematic (upper panel) and actual setup (lower panel) of the Mach–Zehnder interferometer for the modulation characterization of the dual-layer IPS panel. (**b**) Two-section grayscale input image and the observed interference patterns, and the sinusoidal approximation process for the measured interference pattern. (**c**) Full 360-degree phase modulation characteristics of the dual-layer IPS panel.

The gray scale input pairs analyzed in the Jones matrix model are used in the experiment to validate the devised Jones matrix model. We use a Mach–Zehnder Interferometer to measure the modulation characteristics of the designed dual-layer IPS SLM^43^. The experimental setup is shown in Fig. 4a. The collimated light from a solid-state laser (532 nm Lighthouse Sprout-G) is split into signal and reference arms: the dual-layer IPS SLM is placed on the signal arm of the interferometer, and a pair of polarizers are placed on the reference arm to finely control the transmission power of the reference arm. The two light arms of optimally tuned power meet together at the CCD raising an interference pattern that allows us observe the phase delay of the signal arm. Figure 4b shows the double input 2560 × 1600 grayscale images for IPS1 and IPS2, which are composed of the upper and lower parts. The measured interference pattern has two distinctive patterns in its upper and lower sections. The lower interference pattern is the fixed reference and the upper laterally shifts according to the phase delay of the signal beam. The signal part inputs at the gray level from 0 to 255, and the reference part inputs black (gray level of 0), to measure the phase delay of the signal part.

By interpreting the relative lateral shift, we can determine the phase delay of the signal arm accurately. The signal processing of the phase delay extraction is illustrated as a three-step process: measuring the interference pattern, noise removal, and sinusoidal fitting to specify the lateral shift of the interference pattern. Finally, the phase delay is measured by comparing the phase of each part with the fixed reference pattern. Independent modulations of the amplitude and phase of the signal beam are carried out using the pairs of grayscale input values found by the numerical simulations. Firstly, the result verifies that amplitude-independent full 360-deg. phase modulation is possible with the designed device. This is shown on the polar coordinate plot in Fig. 4c, which shows some sampled phase modulation values for comparison with the simulation analysis. The amplitude modulation is almost fixed to a constant value for full 360 (deg.) phase modulation.

Table 2 shows a comparison of some sampled phase modulation values obtained from the simulation and the experimental phase modulation. The actual modulation interval differs to that of the simulation, and there is likely an unignorable difference in the parameters between the simulation parameter and those of the actual fabricated devices. Nevertheless, each input shows a distinct phase delay when reaching full phase modulation and it could be assumed that this error mostly originates from the fluctuation of the experimental system and the inaccuracy of the noise removal process.

**Table 2:** Comparison of phase modulation of dual-layer IPS system between simulations and experiments.

Simulation	Experiment	Simulation	Experiment	Simulation	Experiment
30	27.662	150	145.493	270	269.572
60	53.832	180	170.670	300	298.119
90	85.568	210	199.487	330	327.078
120	117.633	240	236.776		

**Figure 5:**
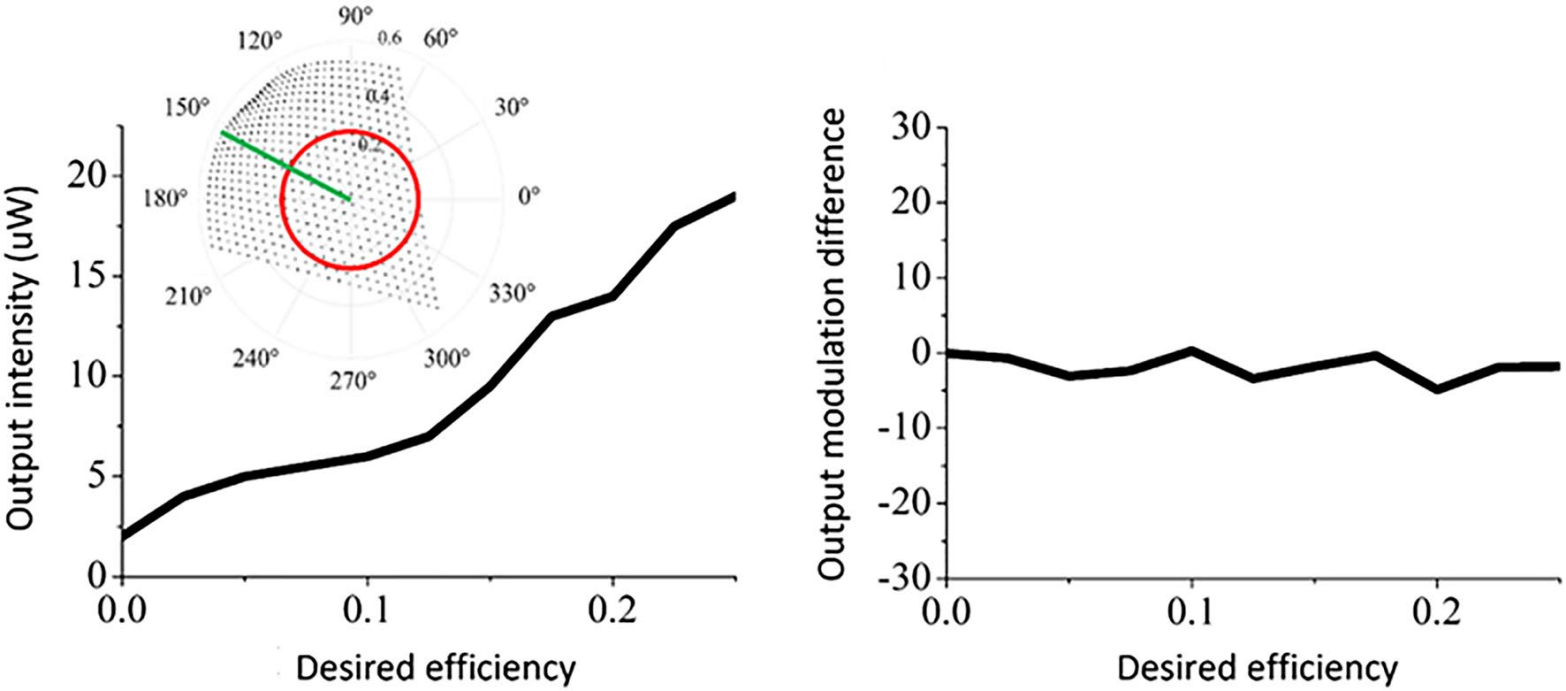
The observed output intensity (left panel) and phase variation (right panel) of the dual-layer IPS-LC SLM at the measurement of amplitude modulation. The inset image shows the straight-lined modulation path of this experiment presenting the amplitude-only modulation capability.

Next, phase-independent amplitude modulation is tested by changing the desired amplitude value, while maintaining the phase. To minimize the unexpected noise factor and observe more accurate results, the amplitude variation should be limited to a maximum range. Although the maximum radius of the modulation circle is set to 0.26 beforehand, the maximum amplitude modulation at a particular phase could exceed that. Therefore, the phase was set to 150 (deg.), as shown in the inset image of Fig. 5, so that the amplitude value could change from 0 to 0.5. In order to change intensity linearly, the desired amplitude value should be quadratically increased. In the experimental results, as shown in the plot in Fig. 5, the phase value (right panel) remains almost constant as the intensity (left panel) increases linearly.

### Generation of complex computer generated hologram

**Figure 6:**
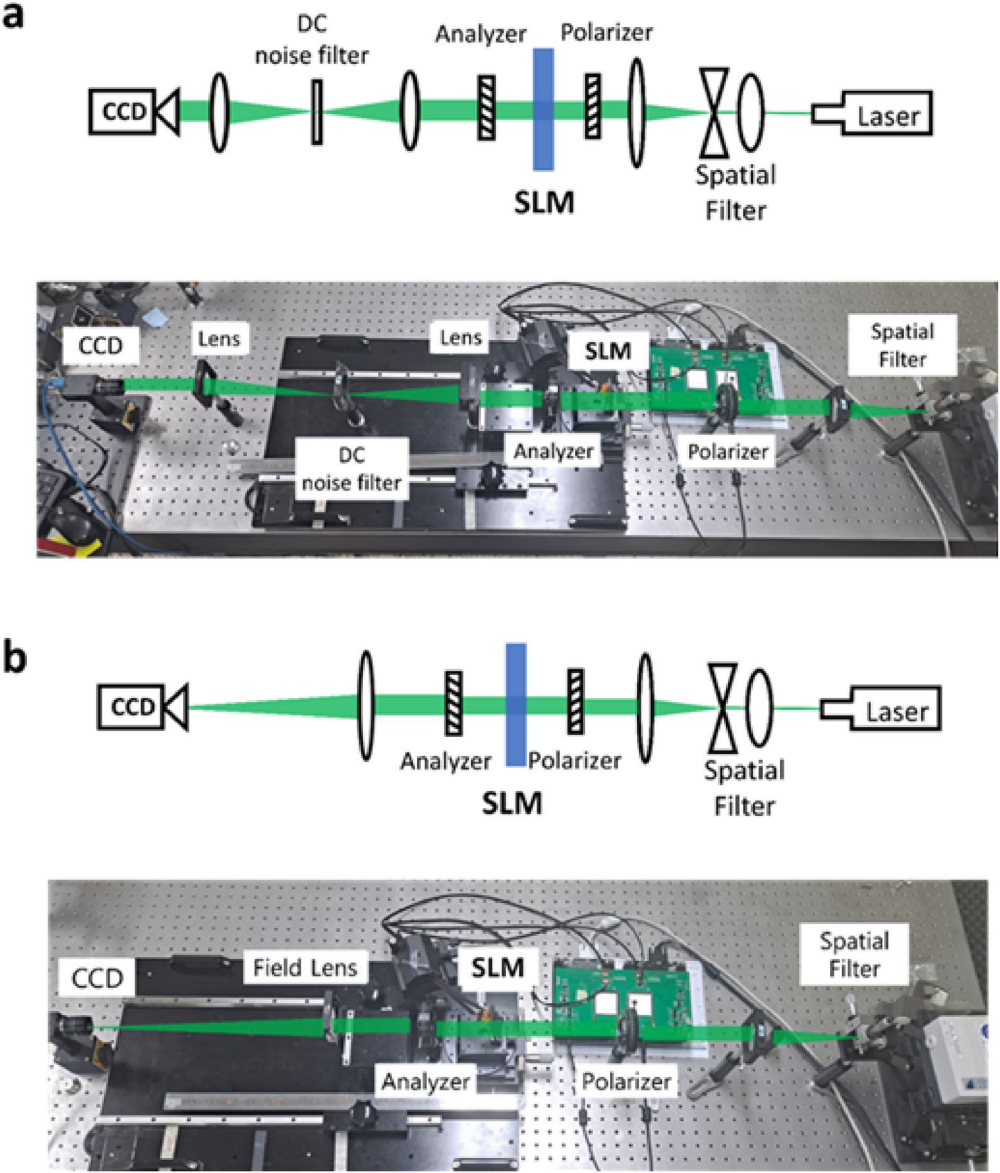
(**a**) Optical Fourier transform test setup and (**b**) 3D holographic image formation test setup. Schematics (upper panel) and implementation (lower panel) of the experimental systems are shown.

As full complex light modulation through dual-layer IPS panel is experimentally proven, a genuine complex computer-generated hologram (CGH) is designed and displayed to further validate the complex spatial light modulation capability of the dual-layer IPS panel. Two types of CGH experiments are set. The former is an experimental comparison of the diffraction pattern synthesis of a complex CGH, an amplitude-only CGH, and a phase-only CGH^44^. Because the elimination of the noises in the diffraction pattern proves the capability of the complex spatial light modulation, we design a simple diffraction pattern and investigate whether the dual-layer IPS panel generates unwanted noises or not. The experimental setup is shown in Fig. 6a. The plane wave goes through the polarizer, the SLM, the analyzer, and a Fourier lens, sequentially, and generates a simple diffraction pattern on the CCD plane. In order to distinctively observe the noise term in the diffraction pattern, we added a DC noise rejection filter between the first Fourier plane and the CCD plane. The second setup shown in Fig. 6b is for a three-dimensional CGH image synthesis of a multi-depth object, allowing examination of the accommodation effect.

**Figure 7:**
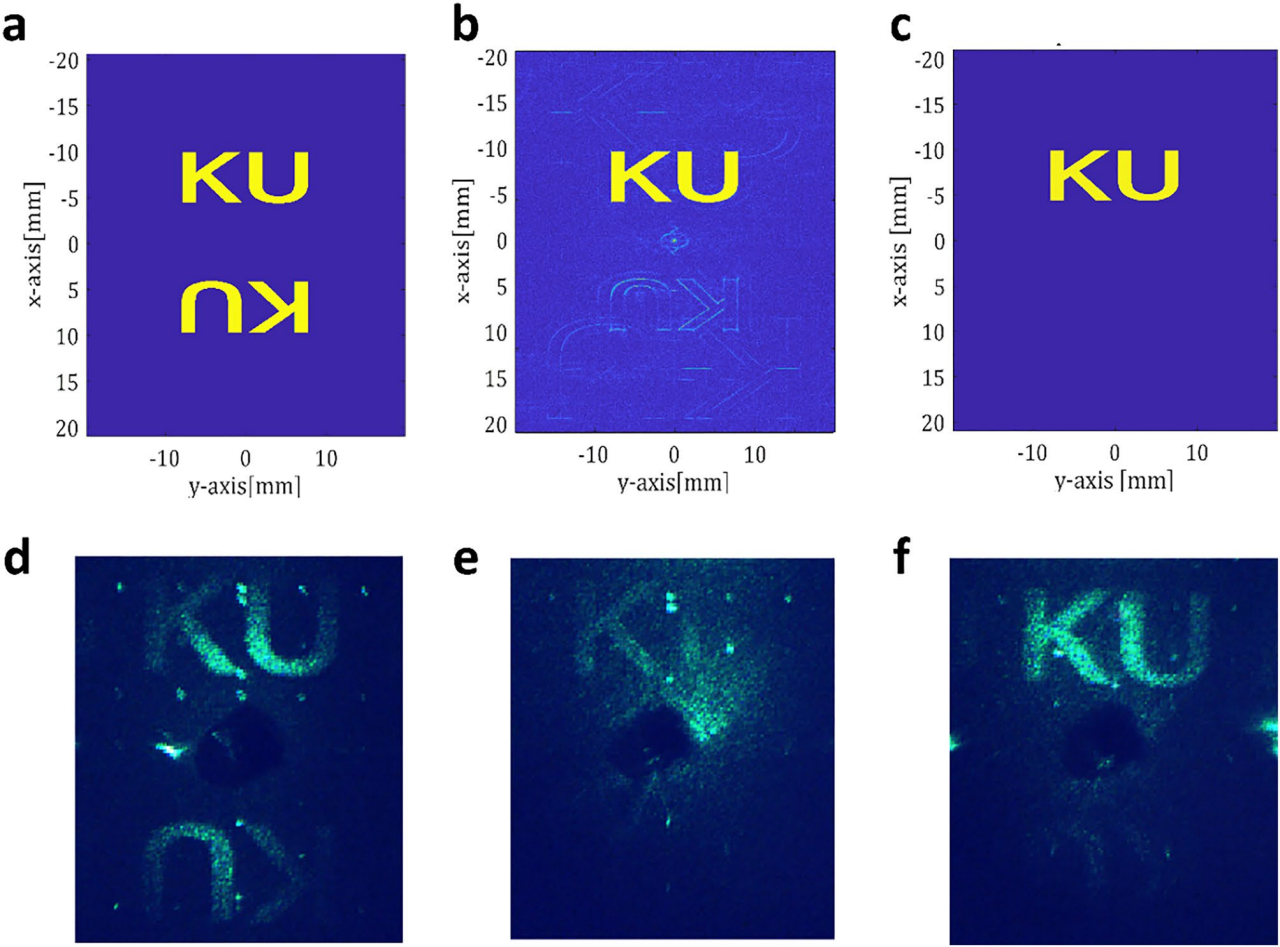
Numerical simulation (**a**–**c**) and experiment (**d**–**f**) for the optical Fourier transform of CGH: (**a**, **d**) the amplitude-only CGH (**b**, **e**) the phase-only CGH, and (**c**, **f**) the complex CGH. A DC noise rejection filter was used to distinctively observe conjugate noise reduction.

Complete complex modulation has been considered a feature of the ultimate holographic 3D display^14,45^. The popular setup is the holographic 3D display based on a single-side band amplitude SLM or phase-only SLM. As aforementioned, inherent optical twin noise has fundamentally hindered the advancement of holographic display technology. Here, complex holographic image field generation is demonstrated with the proposed dual-layer IPS panel. In Fig. 6b, the CCD perceives the optical scene from the SLM without any intermediate optical filter for a demonstration of a true complex holographic display demonstration. For the observation of the diffraction pattern and comparison with other modulation methods, a CGH with the text ‘KU’ is designed. All of the complex light information for the designed far-field CGH is numerically calculated using the angular spectrum method. Then the CGH is processed in three modulation methods: the amplitude modulation, the phase modulation, and the complex modulation. The maximum amplitude of the CGH is normalized to 0.2 in all methods to fit in the dual IPS modulation range. In the amplitude modulation method, only the amplitude information of the calculated CGH is taken and contributes the input pattern. This input image is put into a single IPS panel, which has exactly the same parameters as the dual-layer IPS, and with cross-pole condition. Figure 7a, d shows the simulated and observed far field distribution, respectively, with amplitude modulation. In case of phase modulation method, only the phase information for each pixel is taken. Since a single IPS panel is unable to achieve 360-degree phase modulation, the phase CGH is put into the dual IPS panel. The dual IPS tilt angles for corresponding phase value are obtained and contributes the input pattern. Here, the dual IPS acts as phase-only mode, keeping the amplitude unchanged. Figure 7b, e shows the simulated and observed far field distribution, respectively, with phase modulation. In the complex modulation with dual-layer IPS system, on the other hand, the dual-layer IPS tilt angles for the corresponding complex information are calculated to obtain a pair of grayscale images. This grayscale image pair is then used as the input for the dual-layer IPS panel and the CGH image is observed at the system. Figure 7c, f are the simulated and observed diffraction patterns with the complex modulation, respectively.

In the observation of all methods, the DC noise is suppressed to show the difference more distinctively. As a result, the observed image shows that little conjugate noise is observed at the complex CGH. A faint twin image is shown in the lower region of the field of view, which is most likely caused by a slight modulation error, because the input value is not continuous but a discrete gray level. However, the error can be disregarded compared to the amplitude CGH, which shows distinct conjugate noise, and it can be considered that the modulation capability has been achieved to a sufficient level. In case of the phase CGH, it is well known that it is practically hard to achieve complete black condition and the distinct background noise is observed. This result shows that the proposed dual-layer IPS system is genuinely valid method for the complex CGH.

**Figure 8:**
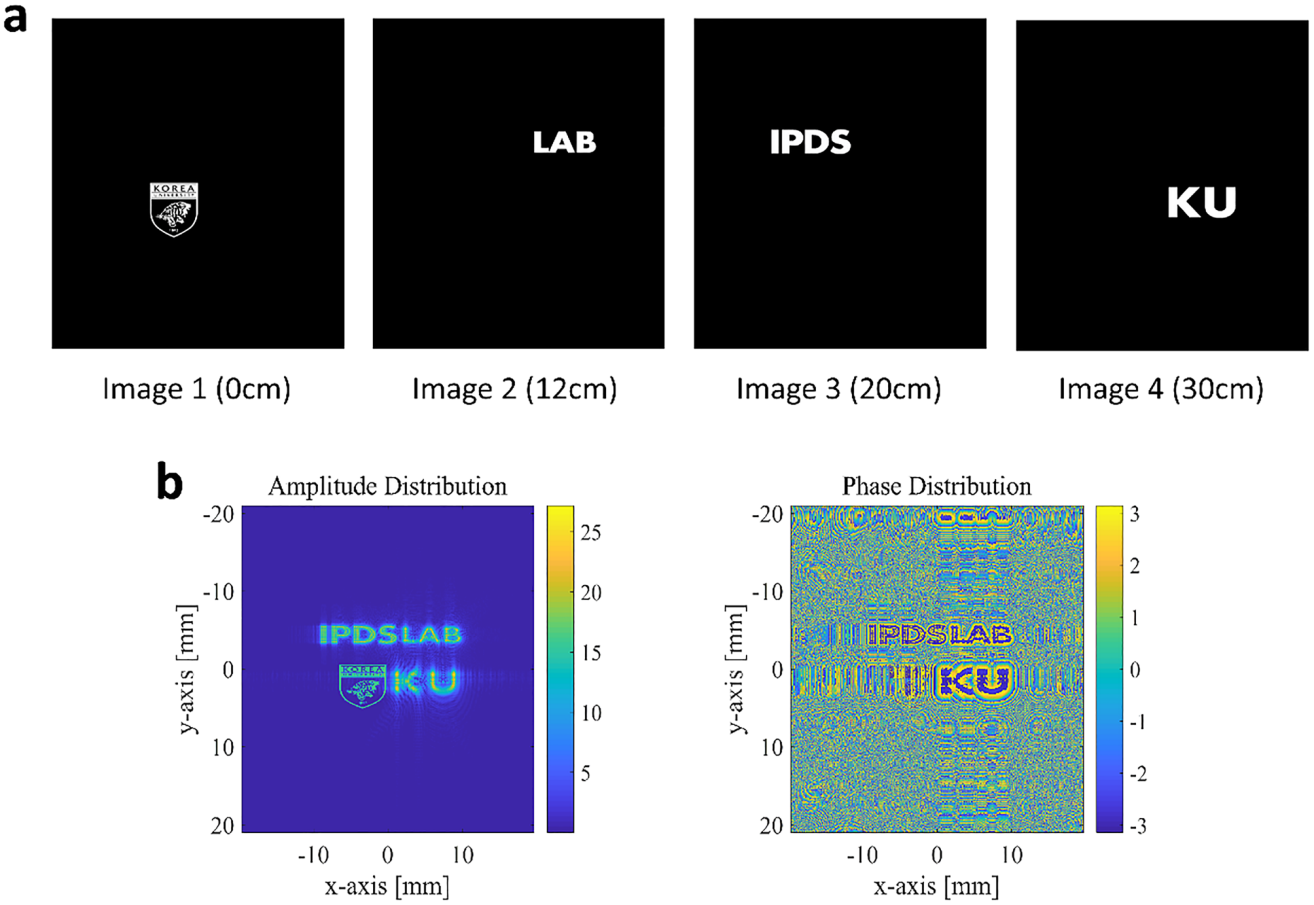
(**a**) Target depth images and the respective focal length of the 4-level CGH. (**b**) The amplitude (left panel) and the phase (right panel) distributions of the calculated CGH.

Next, we designed a CGH representing a four-depth layer object and observe the accommodation effect. Four-layer images are located at the distance of 0 cm, 12 cm, 20 cm, and 30 cm, respectively, from the field lens, as shown in Fig. 8a. The CGH is calculated through the Cascaded Fresnel Transform, which is calculated based on the setup mentioned in Fig. 6b^25,45^. The resulting amplitude and phase distribution of the calculated CGH is presented in Fig. 8b, and the dual IPS complex CGH is generated to achieve the complex distribution. The brief explanation for generating dual IPS complex CGH is in Supplementary Information.

**Figure 9:**
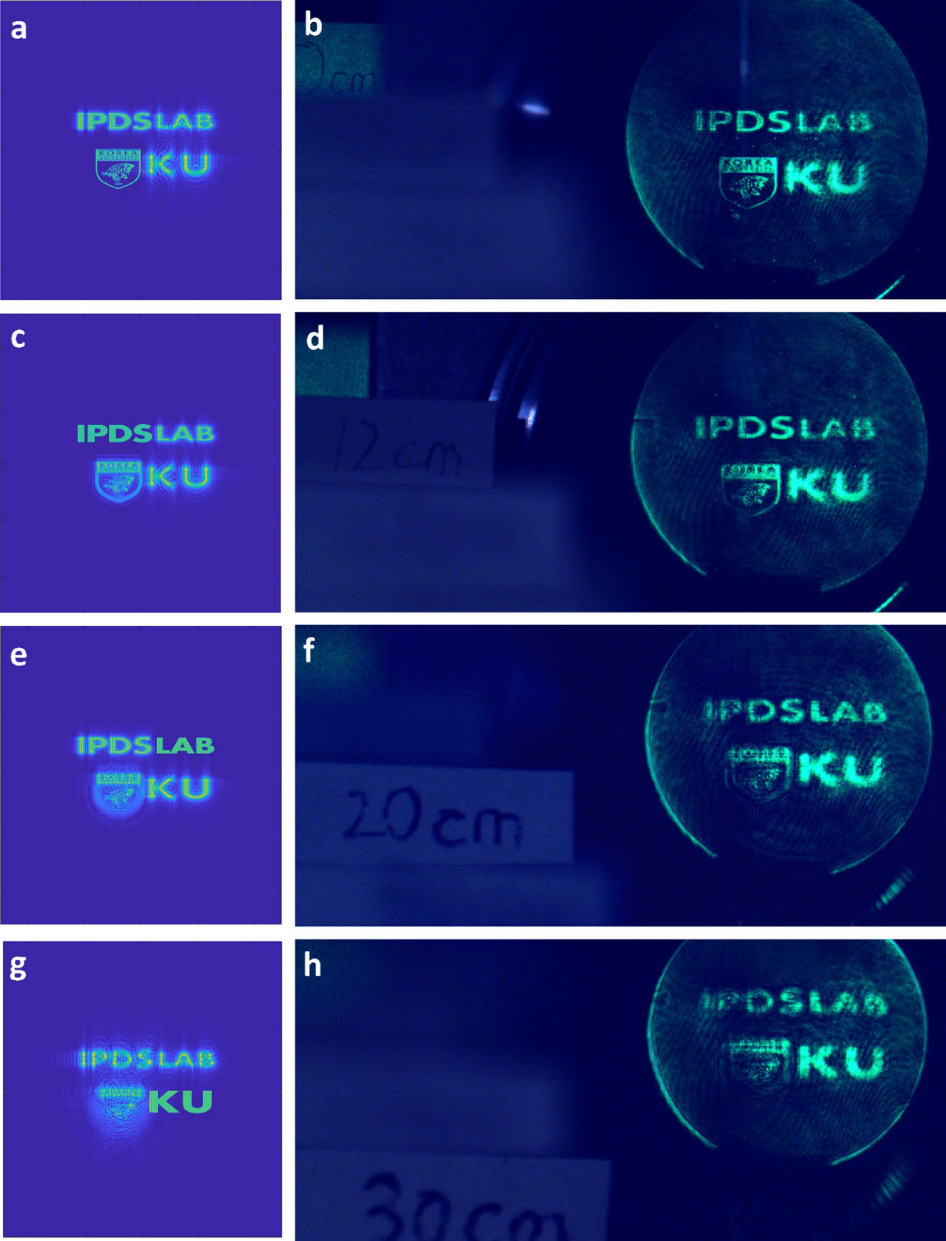
The simulated (left panel) and observed (right panel) holographic image of the designed complex CGH at the foci of 0 cm (**a**, **b**), 12 cm (**c**, **d**), 20 cm (**e**, **f**), and 30 cm (**g**, **h**). An additional video present dynamic operation of the device (Visualization 1).

Figure 9 shows the accommodation effect of the designed CGH image. In the observed results (right panels), the image with the corresponding depth becomes distinct while the other image becomes out of focus and blurred, as expected at the numerical simulations (left panels). No conjugate noise is observed at any depth, and no additional filtering system was employed for DC noise rejection.

In this experiment, the non-diffractive uncontrollable component DC seems to be spread across the background. It is noted that the DC in the first experiment is focused on a point, while in the second experiment, the DC is spread over the background deteriorating the contrast-ratio. With this result, the availability of complex CGHs using the proposed dual-layer IPS system is confirmed. The further DC reduction and transmission efficiency enhancements require further research and development at the architectural design level of the dual-layer IPS panel. As shown at the results, genuine complex CGHs could be designed and observed with the dual-layer IPS LC mode and three-phase complex modulation theory. The background noise is also supposedly originated from the crosstalk occurred between two IPS panel. In the ideal condition, the whole output from the aperture of the first IPS layer enters the corresponding aperture of the second IPS layer, however, the diffraction occurs from the aperture and some portion of the output becomes the crosstalk noise instead of entering the corresponding aperture. The dual-layer IPS panel used in the experiment has 1000 µm gap between two active layers, and therefore diffraction is occurred to cause not only the crosstalk effect but also loss of light efficiency. Using a thinner glass layer to reduce the gap between the active layers could provide better performance. Inserting micro lens array layer between two panels or substituting the glass layer into optic fiber layer could also be considered to decrease the background noise and increase the light efficiency.

The problem of low light efficiency could also be potentially improved both experimentally and theoretically in the way of improving the parameters of the LC display. The IPS-LC display used in the experiment shows a light transmittance of 7.36 percent, which means that the dual-layer IPS would show a light transmittance of 0.54 percent. However, if a display of higher transmittance is used, the improvement in light efficiency would be proportional to the square of the improvement in individual panel transmittance. Here, the point-by-point photoalignment technology^46^ could also be a key to improve the efficiency. Moreover, light efficiency could also be improved by increasing the modulation range. The maximum LC tilt angle of the device could drastically change the modulation range, which is currently restricted to 33.28 (deg.) and the optimal modulation condition could be recalculated as the maximum tilt angle is increased. Simulated results of the modulation conditions with increased maximum tilt angle are shown in Supplementary Information. The value of Γ changes as the wavelength *λ* of the incident light changes. The current study has been carried out under the condition of 532 nm light, but better modulation conditions are probable if the optimal condition is found in the other wavelength range. Moreover, if a certain common modulation range is achieved in red, green, and blue wavelengths with a single device parameter, RGB full color complex modulation would become possible in this range. Applying the light diffraction technique with dual-twist liquid crystal polymer^47^ would also be considerable for realizing full-color light modulation.

## Conclusion

Complex spatial light modulation is becoming a core technology to a vast industrial field. Especially, the transmission-type complex SLM may be advantageous for certain display applications such as holographic AR technology in terms of small-form factor. Furthermore, the complex modulation that covers the entire field is a crucial technology for not only in display but other applications in wave optic systems, which is challenging to achieve with the conventional method. In this paper, the single-pixel complex spatial light modulation capability of the dual-layer IPS-LC mode has been demonstrated both theoretically and experimentally. Full complex light modulation is achieved with the extremely compact system merely consists of a dual-layer IPS devices, a polarizer, and an analyzer. The prototype of the dual-layer IPS LC panel was fabricated through industrial cooperation. The panel is not only demonstrated through simulation but also experimentally using fabricated device, which has proved directly applicable to three-dimensional holographic display technology. Moreover, since the modulation range shown in this paper is not the fundamental limit of this system, it is expected that a simple change of LC panel specification could enhance the complex light modulation characteristics, as shown in Supplementary Information. Along with a further optimization of the LC panel architecture and through a standardization of the precise measurement and inspection method of complex spatial light modulation characteristics, we will enhance the complex modulation efficiency. The achievement of RGB full color complex CGH through the proposed dual-layer IPS LC mode on further scaled-down architecture will be the goal of our next study.

## Supplementary Information

Supplementary Information.

Supplementary Video 1.

## Data Availability

All the data supporting the findings are available from the corresponding author upon reasonable request.
