# Supplementary material for: Complex spatial light modulation capability of a dual layer in-plane switching liquid crystal panel
Source: Sci Rep. 2022 May 18;12:8277. doi: 10.1038/s41598-022-12292-4 (PMC9117259; doi:10.1038/s41598-022-12292-4)
Supplement: Supplementary file 1 — Supplementary Information. [file 41598_2022_12292_MOESM1_ESM.docx]

**Full complex spatial light modulation capability of dual-layered in-plane switching liquid crystal panels**

Seong-Woo Jang^1^, Wonwoo Choi^2^, Soobin Kim^2^, Jonghyun Lee^2^, Sehwan Na^2^, Sangwon Ham^1^, Juseong Park^3^, Hoon Kang^3^, Byeong-Kwon Ju^1^*, and Hwi Kim^2^*

^1^ Display and Nanosystem Laboratory, College of Engineering, Korea University, Seoul, 136-713, Korea

^2^ Department of Electronics and Information Engineering, Korea University, Sejong, 30019, South Korea

^3^LG Display, E2 Block LG Science park, 30, Magokjungang 10-ro, Gangseo-gu, Seoul, Korea

*Corresponding authors: [bkju@korea.ac.kr](mailto:bkju@korea.ac.kr), [hwikim@korea.ac.kr](mailto:hwikim@korea.ac.kr)

1. Complex light modulation theory

The dual IPS system shown in Fig. 1(a) is modeled by Jones Matrix representation [1-4]. The dual IPS system is composed of four components: a polarizer with tilt angle $\theta_{1}$, IPS panel with twist angle , IPS panel with twist angle , and an analyzer with tilt angle $\theta_{2}$. The phase retardation of two IPS panels is the same value $\Gamma$. The Jones matrices of the four terms are denoted by $2\times2$ Jones matrix form. The total Jones matrix of the dual IPS system can be calculated as following:

(S1)

Let us have the following substitution, for convenience sake, $A=\cos\theta_{2}$, $B=\sin\theta_{2}$, $C=\cos\theta_{1}$, $D=\sin\theta_{1}$ and in Eq. (S1). Then the formula becomes

(S2)

Under the x-polarized light incidence, the transmitted electric field is obtained as

(S3)

The x-component $E_{x}$ and y-component $E_{y}$ of the transmitted light are represented, respectively, as

(S4)

And the y-component is

(S5)

The electric field components take the form of three-phase amplitude modulation form of $E_{x\left(y\right)}\sim A_{1}e^{j2\Gamma}+A_{2}e^{j\Gamma}+A_{3}$ enabling the complex light modulation in the dual IPS architecture.

2. Optimal complex light modulation

The condition of complex light modulation can be estimated by the maximum amplitude securing the full-$2\pi$ phase modulation simultaneously.


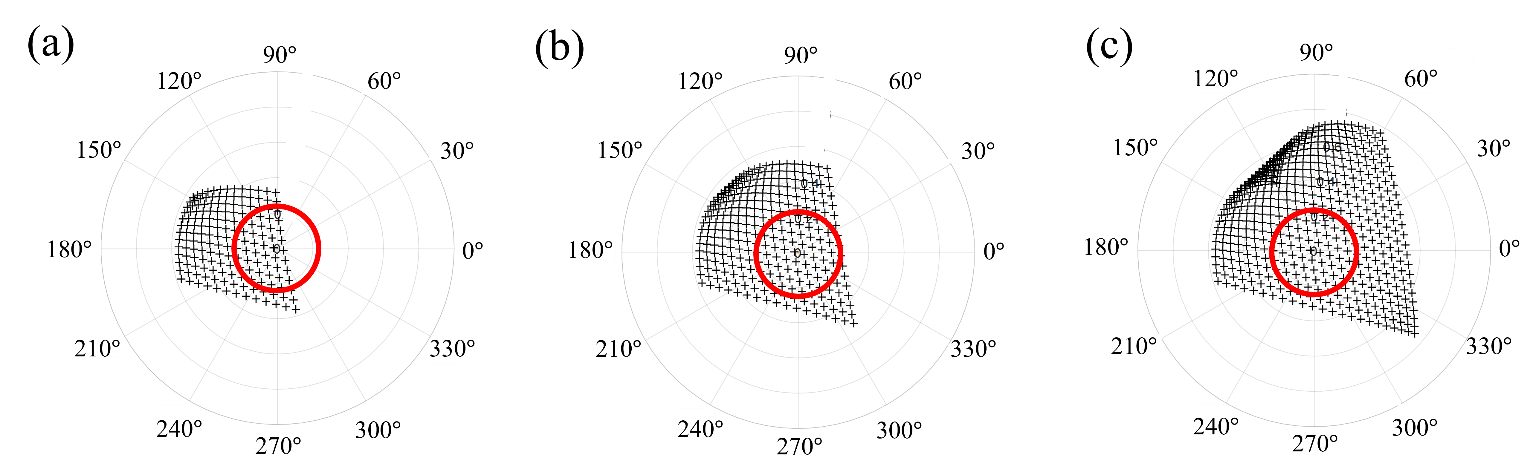


**Fig. S1**. The complex modulation range of the dual IPS system under the condition of $\theta_{1}=0^{\circ}$, $\theta_{2}=125^{\circ}$, at different maximum LC tilt angles, (a) 25 (deg.), (b) 33.28 (deg.), and (c) 45 (deg.). The red circles indicate 0.26 amplitude in all figures.

The optimal condition for complex light modulation may vary with the system parameters$\theta_{1}$, , , $\theta_{2}$ and $\Gamma$. Particularly, we have found that the modulation range changes dramatically according to the maximum IPS tilt angle change. Fig. S1 shows the modulation ranges under the condition of$\theta_{1}=0$(deg.), $\theta_{2}=125$(deg.), when the maximum LC tilt angle is 25(deg.), 33.28 (deg.) (b, current condition) and 45 (deg.) (c). The other system parameters are identical as the main text. As shown in Fig. S1, the increased maximum LC tilt angle leads to the enhanced modulation range. Moreover, when other system parameters change, the optimal modulation condition also can change leading to a further enhancement of complex modulation range, i.e. complex modulation efficiency. Numerical optimization finds some design examples for the new system parameters. Fig. S2 shows the optimal modulation characteristics of the dual IPS system with the newly found $\theta_{1}$ and$\theta_{2}$ when the maximum tilt angles for the left and right cases are assumed to be 45 (deg.) and 90 (deg.), respectively. In each condition, the full complex modulation under the amplitude of 0.44 and 0.6 is achieved, respectively. To conclude, the modulation range shown at the research is not the fundamental limit of the system, and the other optimal conditions could be found for the different cell gap or incident wavelength.


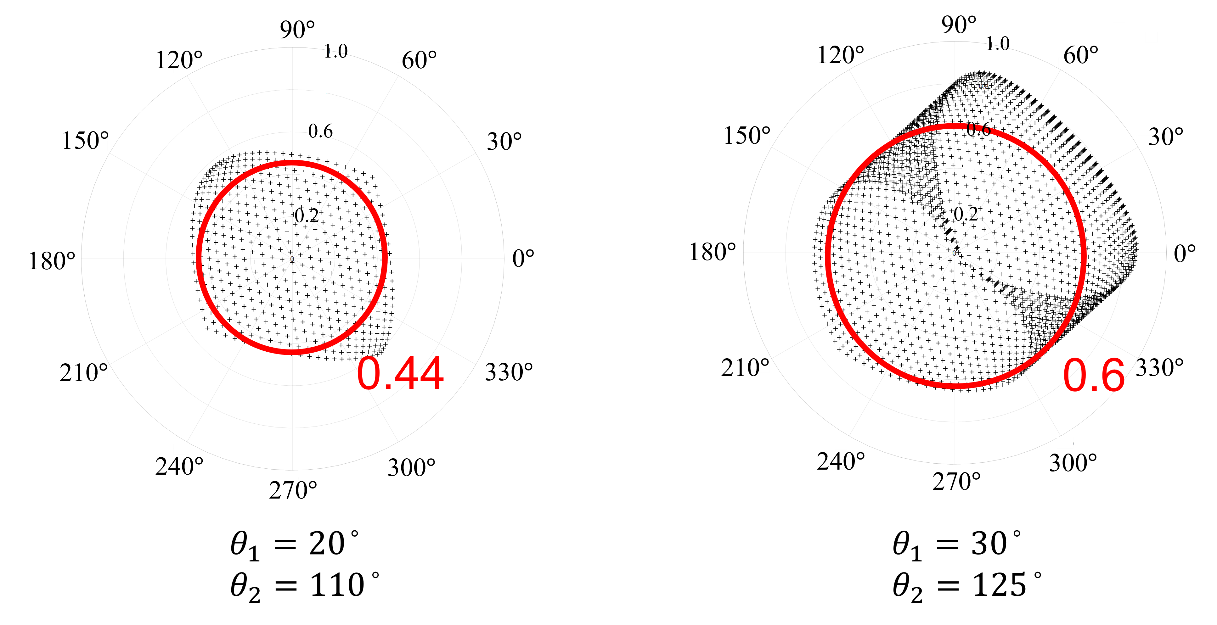


**Fig. S2.** The optimal light modulation characteristics and polarizer conditions of the dual IPS system when the maximum LC tilt angle is 45 (deg.)(left) and 90 (deg.) (right).

3. Generation of the dual IPS complex CGH


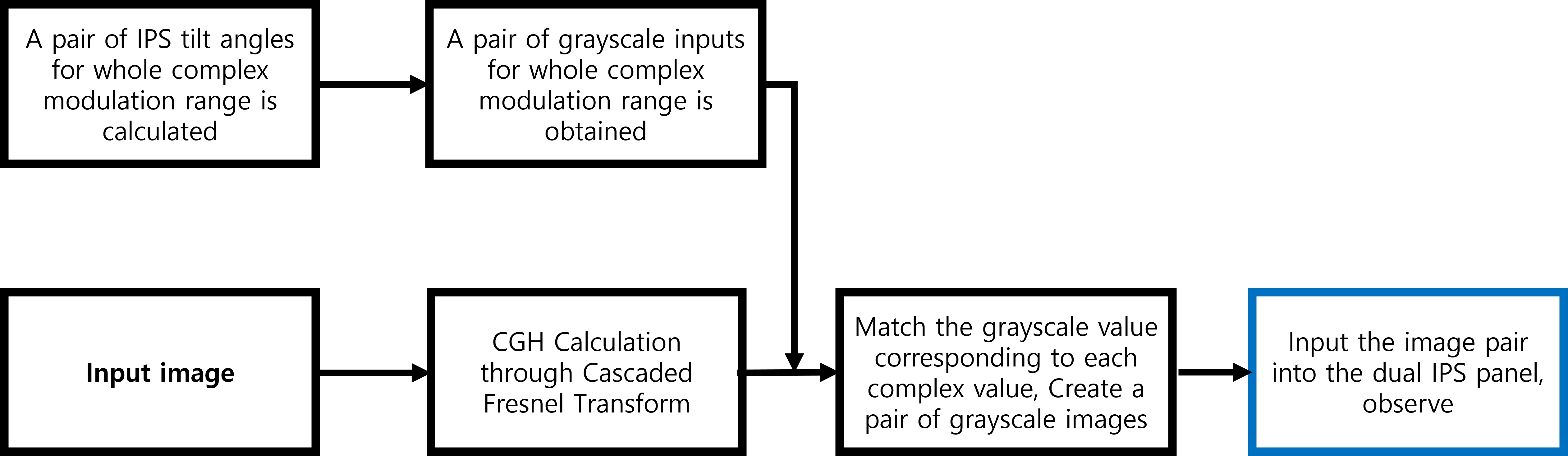


**Fig. S3.** Algorithm for the dual IPS complex CGH.

Fig. S3 shows the flowchart for generating a dual IPS complex CGH. First, IPS tilt angles corresponding the whole complex modulation range is obtained through the Jones Matrix calculation as mentioned beforehand. Since the grayscale values for whole IPS tilt angles are known, the pair of grayscale inputs for whole complex modulation range could be obtained and built a look-up table for fast addressing. Then, CGH is calculated through Cascaded Fresnel Transform [5-6] and a matrix of complex values corresponding to each pixel is obtained. With the look-up table, the grayscale value for corresponding complex value could be found quickly and contribute a pair of grayscale images. These images are input to the dual IPS panel, and the CGH is observed.

**References**

1. C. Soutar, and K. H. Lu,"Determination of the physical-properties of an arbitrary twisted-nematic liquid-crystal cell," Opt. Eng. **33**, 2704-2712 (1994).

2. J. E. Wolfe, and R. A. Chipman,"Polarimetric characterization of liquid-crystal-on-silicon panels," Appl. Opt. **45**, 1688-1703 (2006).

3. N. K. Soni, A. S. Somkuwar, and R. K. Singh, *Jones matrix imaging for transparent and anisotropic sample* (SPIE, 2015).

4. I. Moreno, J. A. Davis, K. G. D'Nelly, and D. B. Allison,"Transmission and phase measurement for polarization eigenvectors in twisted-nematic liquid crystal spatial light modulators," Opt. Eng. **37**, 3048-3052 (1998).

5. J. Roh, K. Kim, E. Moon, S. Kim, B. Yang, J. Hahn, and H. Kim, "Full-color holographic projection display system featuring an achromatic Fourier filter," Opt. Express **25**, 14774-14782 (2017).

6. D. Im, E. Moon, Y. Park, D. Lee, J. Hahn, and H. Kim, "Phase-regularized polygon computer-generated holograms," Opt. Lett. **39**, 3642-3645 (2014).
